# Towards population screening for Cerebral Visual Impairment: Validity of the Five Questions and the CVI Questionnaire

**DOI:** 10.1371/journal.pone.0214290

**Published:** 2019-03-26

**Authors:** Fiona Gorrie, Karen Goodall, Robert Rush, John Ravenscroft

**Affiliations:** 1 School of Health in Social Sciences, Department of Clinical Psychology, The University of Edinburgh, Edinburgh, Scotland; 2 School of Health Sciences, Division of Dietetics, Nutrition & Biological Sciences, Physiotherapy, Podiatry & Radiography, Queen Margaret University, Edinburgh, Scotland; 3 The Scottish Sensory Centre, The University of Edinburgh, Edinburgh, Scotland; Universiti Sains Malaysia, MALAYSIA

## Abstract

**Introduction:**

Cerebral Visual Impairment (CVI) is the most common cause of visual impairment in children in the developed world and appears to be more prevalent in children with additional support needs (ASN). There is an urgent need for routine screening for CVI, particularly in children with ASN, however, current screening questionnaires for CVI have limited validation. The aim of this study was to evaluate two screening tools: the Five Questions and the CVI Questionnaire. Additionally, the distribution of CVI across neurodevelopmental disorders is unknown. This too was investigated.

**Methods:**

An online survey was completed by 535 parents. The survey was advertised via social media, CVI websites and parent email systems of four schools. The survey comprised of the Five Questions, the CVI Questionnaire and additional questions regarding the child’s diagnoses. Whether or not a child had a diagnosis of CVI and/or additional neurodevelopmental disorders was based on parental report.

**Results:**

Based on parent reports, both the screening tools accurately screened for CVI diagnoses in children. The Five Questions and the CVI Questionnaire have construct validity (as determined through factor analysis), high internal consistency (as determined by Cronbach’s alpha) and convergent validity (as determined by correlation analysis of the raw scores of each questionnaire). This study also highlights that among children with neurodevelopmental disorders, a large proportion have parent-reported CVI (23%-39%) and potential CVI (6.59–22.53%; as identified by the questionnaires).

**Conclusion:**

The current study demonstrates that the Five Questions and CVI Questionnaire have good convergent validity, internal consistency and a reliable factor structure and may therefore be suitable as screening tools. The study also highlights that reported or potential CVI is evident in a large proportion of children with neurodevelopmental disorders.

## Introduction

Vision impairment in early life can have a significant negative impact on quality of life [1] and can impose substantial constraints on later development, in areas such as integration of input from other senses, motor competence, language and cognitive concepts [2]. Visual impairment can arise from damage anywhere along the visual pathway with traditional approaches focusing on dysfunction of the eye and the optic nerves (ocular visual impairment, OVI). Impairment may also be the result, however, of damage to structures lying beyond the eye itself. Cortical/Cerebral Visual Impairment (CVI) refers to damage to the visual pathways often known as the dorsal and ventral pathways, and associated structures that occurs during early perinatal development. The term ‘Cortical Visual Impairment’ was originally coined to refer to visual impairment of non-ocular origin, with an underlying assumption of damage to early visual cortical areas [3]. As it became clear that CVI was frequently associated with damage to areas beyond the early visual cortex, including higher order processing areas of the cortex, the term ‘cerebral’ has been more widely adopted [4]. A systematic review of terminology and definitions of CVI suggests that the most parsimonious definition is *a verifiable visual dysfunction which cannot be attributed to disorders of the anterior visual pathways or any potentially co-occurring ocular impairment* [5].

CVI is the primary cause of congenital visual impairment in children in the economically developed world [6, 7]. In the United Kingdom, CVI affects 40–48% of children with impaired vision [8, 9]. Prevalence rates of CVI continue to rise in developed countries, partly as a result of neonatal care advancement, leading to better survival rates in preterm infants [10]. Accordingly, CVI has been associated with low birth weight (<2500 g) [8] and prematurity [11, 12, 13]. Prevalence rates between 33% and 58% have also been noted in children with cerebral palsy (CP), hydrocephaly and periventricular white matter injury (PVL) [12–16], supporting the link between early brain insult and CVI. Several studies, based on optometrist/ophthalmologist assessment have also reported an increased risk of visual impairment in children with neurodevelopmental disorders [2, 17–22], although evidence on associations between specific types of neurodevelopmental disorders and CVI is limited.

The rising prevalence rate of CVI, along with evidence of its potential impact on later development, suggests that CVI should be considered as a pressing public health issue e.g. [23]. Although research has focused traditionally on outcomes related to ocular impairment, an accumulating body of evidence suggest that there are significant sequelae associated with CVI, including motor and cognitive and language development [24–26].

The absence of ocular pathology alongside a relative lack of awareness of the condition in healthcare providers means that CVI is likely to go undetected in some children and in others difficulties may be misdiagnosed as a disorder that is behavioural or psychological in nature [27–30]. Furthermore, Martin et al. [31] suggest that underlying differences in brain structural connectivity mean that strategies suitable for individuals with ocular impairment will not be effective for those with CVI. As there is evidence that early identification and intervention can lead to developmental gains [32], screening is particularly warranted in children with Additional Support Needs (ASN- note that this terminology is used in place of ‘special needs’ in Scotland); [20, 30, 33].

Expert testing for CVI is likely to be costly and not feasible at the population level. In addition, few ophthalmologists are confident in the clinical markers of CVI [34] and there are a limited number of clinicians who can diagnose CVI, for example in Scotland there are only three [35]. Parent-rated screening questionnaires are therefore a potentially cost-effective means of screening larger groups of at-risk children. They can reach large populations of children with relative ease, exclude those unlikely to have CVI and therefore reduce the numbers who warrant clinical assessment [36]. Several CVI screening questionnaires exist which have been developed by clinicians experienced in diagnosing CVI, e.g. [36–41], however some have not been validated e.g. [37, 40], whilst four [36, 38, 39, 41] have limited predictive validation through establishing cut-off points and comparing rates of screened positives on the questionnaire against confirmed CVI diagnoses. The present study focuses on the CVI Questionnaire [39] and the Five Questions [38].

### The current study

The CVI Questionnaire has been recommended as a tool which can assist with the diagnosis of CVI [42], however it has been rated as having poor structural validity and failing to assess internal consistency, reliability, or measurement error [43]. Whilst it is claimed that the Five Questions are predictive of CVI [38], there is no peer reviewed validation of the Five Questions. Construct validity has not been assessed through Factor Analysis for either questionnaire, meaning that further validation of the questionnaires is required [36] before whole population screening studies can be carried out.

The primary aim of the current study is to assess the internal validity and construct validity of the aforementioned questionnaires. A second aim is to assess the performance of the questionnaires in predicting diagnosis in children whose parents report a CVI diagnosis. As these questionnaires contain some overlapping items, convergent validity will assessed by examining the correlation between the questionnaires. Finally, the distribution of potential CVI cases across neurodevelopmental disorders will be assessed.

## Methods

### Materials

This study was based on a survey which consisted the following:

1) A short demographics section (S1 File) asked for information on the child’s gender, age and any disability or diagnosis. Parents were also asked to indicate whether the child had a diagnosis of CVI. Information on how the diagnosis was gained was not requested.
2) *The Five Questions* [38] were originally derived from a 50 item CVI Inventory [44]. The full CVI Inventory [44] is used following a CVI diagnosis to ascertain the exact nature of the CVI e.g. dorsal and/or ventral stream impairment. The Five Questions refer to difficulties impaired in (Q3-5), or commonly associated with (Q1-2) Dorsal Stream Dysfunction. Parents were instructed to indicate, on a 5-point Likert Scale, whether their child ‘always’, ‘often’, ‘sometimes’, ‘rarely’ or ‘never’ struggles with the described tasks. To derive the Five Questions from the full inventory, 40 children with CVI and 150+ controls without were tested [37]. For the Five Questions, Dutton et al. [37] reported that parents of children with CVI consistently respond ‘often’ or ‘always’ to 3 or more questions, while parents of children without CVI respond affirmatively to a maximum of one item. Cronbach’s alpha has not been calculated for the Five Questions. However, the full inventory has 7 domains, across domains the Cronbach’s alpha is between 0.71 and 0.82, in children with CVI aged 5–17 [44]. Internal reliability of the full inventory has also been tested on populations in India with Autism Spectrum Disorder (ASD), CP and Epilepsy: Cronbach’s alpha = 0.93 [45]. Furthermore, the full inventory is recommended for use with those with mild intellectual disability, ID, [46].
3) *The CVI Questionnaire* [39] is 46 item screening questionnaire, which lists items that children with CVI have difficulty with. Parents are requested to tick items which apply to their child. The questionnaire used expert opinion to create six constructs of visual attitude, ventral stream, dorsal stream, complex problems, other senses and associated characteristics. The original questionnaire was tested on children (aged 3–17) with and without CVI, with a range of disabilities such as mild and moderate ID, Epilepsy, CP and ASD. All children had a developmental age of between 3 and 6 years. Children are predicted to test positive for CVI if they receive a sum score greater than or equivalent to the cut-off (at least one item ticked in at least 4 of the 6 constructs). Under this cut-off, the sensitivity (probability that the measure will correctly detect those with CVI) is 83.3% and specificity (the likelihood that those without CVI will have a negative test result) is 47.5%. Therefore, it appears a good measure for predicting CVI in children [47]. Cronbach’s Alpha was not reported by the authors.

### Procedure

Parents of Typically Developing (TD) children and children with ASN aged 5–18 were invited to complete the online survey. The survey was advertised via social media, CVI websites and the parent email systems of four schools; two mainstream primary schools and two specialist provisions in a large local authority within Scotland. Parents were unable to begin the survey until they had ticked a box indicating that they had read the information sheet provided and given consent. University ethical approval was obtained from the named institutional review board: University of Edinburgh, School of Health in Social Sciences (approval number CLIN463). Local Education Authority (Edinburgh City Council) approval was also obtained.

### Participants

Five hundred and forty four participants completed the survey; 9 data sets were removed as the children were not within the specified age range (aged 5–18). The final data set comprised 535 participants (Child Age x^-^ = 9.99, σ = 3.47, Child Age Range = 5.01–18.99; Child Sex = Male 60%: Female 40%). Sixty three percent of participants attended a mainstream school, 15% attended a mainstream school with a classroom assistant, 4% were educated in a ‘special class’ within a mainstream school, 14% attended special school and 4% were home schooled. The majority of the sample (76%) were UK residents; the remainder resided in the USA (15%) or other countries.

### Analytical strategy

Parents were asked the question, “Does your child have CVI?” They were not requested to provide information on whether this had been formally diagnosed. Affirmative responses are referred to as ‘reported CVI’. ‘Potential CVI’ refers to those who meet the cut-off score for CVI according to the screening questionnaires.

The ability of both the CVI Questionnaire and Five Questions to predict CVI in children with a reported CVI diagnosis was evaluated using binary logistic regression. The convergent validity of the screening measures was also investigated via correlation analysis of their raw scores.

To evaluate construct validity, Exploratory Factor Analysis (EFA) and not Principal Component Analysis (PCA) was conducted because the central assumption of PCA is that all variance is shared variance [48]. This is theoretically implausible because there is additional error variance to consider [48, 49]. EFA on the other hand, accounts for this: it only evaluates the shared variance, with the remaining variance analysed as specific error variance [48, 49]. For the Five Questions, EFA was conducted using SPSS Version 23.

The CVI Questionnaire has a dichotomous question format. Due to the reliance on a correlation matrix, EFA carried out on dichotomous variables in SPSS is at risk of outputting “*difficulty factors*” which are based on a similar distribution, and not on similarity of content [50, 51]. The EFA on the CVI Questionnaire was conducted in R Studio, allowing a polychoric correlation matrix to be created from the dichotomous variables.

For factors to be retained, <50% of residual correlations had to be > |0.05| [52]. Additionally, each factor had to have at least 3 variables with a primary loading of at least 0.3 to remain in the analysis [53]. Retained factors had to explain > = 25% of the variance [52].

### Power analysis

For a binary logistic regression with one predictor, a minimum N of 100 is required [54]. For factor analysis on health questionnaires, a sample size seven times the number of items in the questionnaire, with a minimum N of 100, is recommended [55], cited in [56]. The largest questionnaire in this study (the CVI Questionnaire) has 46 items. Therefore, 329 participants were required.

## Results

### Descriptives

Table 1 displays the percentage of children with different diagnoses in the sample. Nineteen percent of parents (N = 104) confirmed that their child had a diagnosis of CVI. Sixty six percent of parents reported that their child had at least one diagnosis. Many respondents indicated that their child had multiple diagnoses. In these cases, children are included in multiple groups (e.g. both ASD and Attention Deficit Hyperactivity Disorder, ADHD).

**Table 1:** Diagnoses in sample.

	N	% of Sample
No Diagnosis	181	34
Autism Spectrum Disorder (ASD)	147	28
Attention Deficit Hyperactivity Disorder (ADHD)	87	16
Dyscalculia	39	7
Dyslexia	91	17
Dyspraxia	71	13
Ocular Visual Impairment (OVI)	63	12
CVI	104	19
Hearing Impairment	32	6
Auditory Processing Disorder (APD)	99	19
Intellectual Disability (ID)	167	31
Hydrocephaly	11	2
Epilepsy	37	7
Cerebral Palsy (CP)	32	6
Periventricular White Matter Injury (PVL)	26	5

Note: for definitions of each of the above diagnoses see the attached survey in S1 File.

#### Distribution of reported CVI across diagnostic groups

Table 2 highlights that between 23%-39% of children with a neurodevelopmental disorder have comorbid CVI (according to parental report).

**Table 2:** Percentage of reported CVI by diagnosis.

	% reported per CVI diagnostic group
Autism Spectrum Disorder (ASD)	24
Attention Deficit Hyperactivity Disorder (ADHD)	23
Dyscalculia	36
Dyslexia	26
Dyspraxia	32
Ocular Visual Impairment (OVI)	27
Hearing Impairment	38
Auditory Processing Disorder (APD)	32
Intellectual Disability (ID)	39
Hydrocephaly	82
Epilepsy	54
Cerebral Palsy (CP)	91
Periventricular White Matter Injury (PVL)	92

### Factor analyses

#### Five questions

Loess lines indicated that the variables were linearly related (S1 Fig). Visual inspection of the Histograms, and significant K-S tests, Kurtosis and Skewness values indicated that the variables were positively skewed and not normally distributed (S1 Fig). This is unsurprising as CVI is relatively uncommon. Furthermore, EFA is robust to deviations from normality [50]. Initial correlations (0.55–0.88) and communalities (0.45–0.72) were >0.3 and <0.9, indicating that they are not correlated too weakly or strongly with each other. A KMO test statistic of 0.85 was above the recommended threshold [57] and a significant Bartlett’s Test of Sphericity indicated that all correlations were >0, *X*_*2*_(10) = 1687.76, p<0.001 [57]. Therefore, requirements of EFA were met and no variables removed at this stage.

Twenty percent of the residuals were >|0.5|. Parallel Analysis returned 1 Factor. This aligned with the Scree Plot (S2 Fig). The extracted factor explains 71.5% of the variance. S1 Table summarises this factor and highlights that all 5 variables load onto the one factor, with primary loadings of >0.3. As only one factor was identified, no rotation was carried out. As all variables load onto the one factor, which aligns with the hypothesised construct (dorsal stream functions) this questionnaire has good construct validity [58]. A Cronbach’s Alpha of 0.90 suggests a high level of internal consistency for the questionnaire [59].

#### CVI questionnaire

The 46 items of the CVI questionnaire were subjected to PCA. Firstly, the suitability of the data for factor analysis was assessed. The correlation matrix revealed that all coefficients were 0.3 and above, and below 0.9. The KMO value was 0.79, exceeding the recommended value of 0.6 [57] and Bartlett’s Test of Sphericity reached statistical significance.

PCA with oblique (promax) rotation revealed the presence of five factors with eigenvalues exceeding 1, explaining 23%, 13%, 7%, 5% and 4% of the variance respectively. These accounted for 52% of the variance overall. Inspection of the screeplot (S3 Fig) revealed a clear break after the fifth component. This was further supported by Parallel Analysis which showed five components with eigenvalues exceeding the corresponding criterion values for a randomly generated matric of the same size (46 variables x 535 respondents). All factors had at least three variables with a primary loading of >0.3. Any variables with loadings <0.3 were removed from the factors. The variable “*often stares at light sources”* did not load >0.3 onto any of the factors and was removed. S2 Table indicates the factor loadings after rotation.

Following from Ortibus et al. [39], we have named the factors as follows; F1-Complex Neurological Problems, F2-Dorsal and Ventral Stream Functions, F3-Visual Attention, F4-Influence of a Familiar Environment on Vision and F5- Processing in Multi-Tasking Activities. S2 Table highlights that some variables loaded onto multiple factors. For example, many load relatively equally onto two factors, such as “*cannot play memory games*” (F2, *r* = 0.49, and F5, *r =* 0.44) and “*has no interest for complex pictures*” (F1, *r =* 0.40 and F2, *r =* 0.44). However, the variables with cross loadings were not removed as it was deemed conceptually viable that they were related to each factor.

To summarise, the factors in the current solution reflect constructs associated with CVI and are similar to those proposed by Ortibus et al. [39], demonstrating good construct validity [58]. Cronbach’s Alpha for the whole CVI Questionnaire is 0.94, indicating a high level of internal consistency [59]. Additionally, all factors have an acceptable internal consistency (α > 0.7 and <0.94, S2 Table; 59).

### Criterion reliability: Binary logistic regression of a reported CVI diagnosis onto questionnaire scores

Binary Logistic Regression was conducted to investigate whether the questionnaires had acceptable criterion validity; in other words how accurately they identify the children with a reported CVI diagnosis. The independent variable was whether or not each child met the predefined cut-off scores for each questionnaire (see methods section), and thus were identified by the questionnaires as having CVI (or not). The dependent variable was whether or not parents reported a CVI diagnosis.

#### Five questions

**Fig 1:**
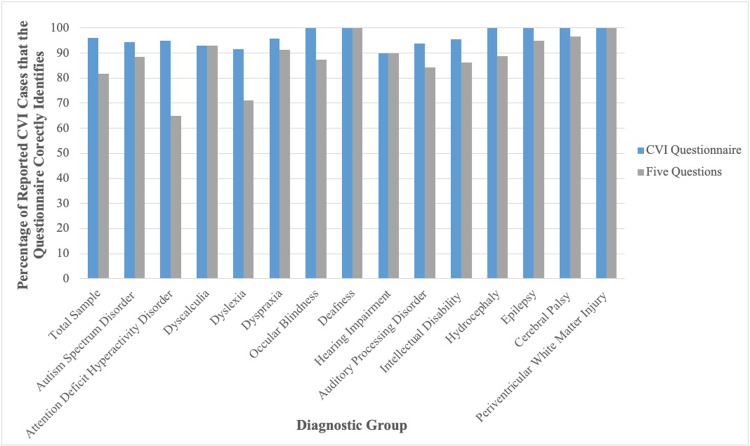
Comparison of the CVI questionnaire and Five Questions at determining children with parental-reported CVI who meet the cut-off for CVI across diagnostic groups.

Fig 1 illustrates that the Five Questions had a sensitivity value of 81.7%. Additionally, its specificity value was 87.2%. Twelve point eight percent of those who selected ‘no’ to a CVI diagnosis were identified by the Five Questions as having CVI.

Table 3 shows a logistic regression which highlights that meeting the cut-off for CVI on the Five Questions (or not) is a significant predictor of whether a child has a reported CVI diagnosis (or not). The model was adjusted for age. Gender was not included as a covariate as it was unrelated to reported diagnosis in a univariate analysis (Odds Ratio = 0.84, 95% CI 0.54, 1.29, p = 0.42).

**Table 3:** Logistic regression of reported CVI diagnosis onto Five Questions cut-off scores.

	p	Odds Ratio	95% CI for Odd’s Ratio
Five Questions Cut-Off	0.00	37.43	20.13–69.60
Age	0.00	0.84	0.77–0.91

The positive odds ratio indicates that children who meet the cut-off on the Five Questions, are 37.43 times more likely to have a reported CVI diagnosis than those below the cut-off. This is further supported by the fact that the lower and upper bound CIs for the odds ratio are >1 and do not cross 1 [60].

#### CVI questionnaire

Fig 1 highlights that the CVI Questionnaire had a sensitivity value of 96.2%. It also had a specificity value of 61.5%. Thirty eight point five percent of those who did not have a reported CVI diagnosis met the cut-off for CVI on the CVI Questionnaire.

Table 4 shows a logistic regression which highlights that meeting the cut-off for CVI on the CVI Questionnaire is a significant predictor of whether or not a child has a reported CVI diagnosis. The model was adjusted for age. Gender was not included as a covariate as it was unrelated to reported diagnosis in a univariate analysis (Odds Ratio = 0.84, 95% CI 0.54, 1.29, p = 0.42).

**Table 4:** Logistic regression of reported CVI diagnosis onto the CVI questionnaire cut-off scores.

	p	Odds Ratio	95% CI for Odd’s Ratio
CVI Questionnaire Cut-Off	0.00	41.96	15.06–116.90
Age	0.001	0.88	0.82–0.95

The positive odds ratio indicates that children who meet the cut-off on the CVI.

Questionnaire, have an almost 42 fold increase in odds over those below cut-off of having a reported CVI diagnosis. This is further substantiated by the fact that the lower and upper bound CIs for the odds ratio are >1 and do not cross 1 [60].

### Sensitivity of questionnaires across diagnostic groups

Fig 1 indicates that the questionnaires both appear relatively accurate at identifying those with reported CVI, across all diagnostic groups. It is apparent that both the Five Questions and CVI Questionnaire predict CVI, with sensitivity values >81.7% and specificity values >61.5%.

### Convergent validity of questionnaires

To explore convergent validity, the relationship between the raw scores of the questionnaires was investigated. Neither of the questionnaires indicated how a raw score should be calculated. Therefore, for the Five Questions and the CVI Questionnaire, the raw score was calculated to align with cut-off scores. For the Five Questions, the raw score is the number of questions which a respondent marked ‘often/always’ to (Maximum Raw Score = 5). For the CVI Questionnaire, the raw score was calculated as the number of sections which a participant gave at least one positive response (Maximum = 6). For both questionnaires, the higher the raw score, the higher the likelihood of having CVI.

K-S tests were significant for the raw scores of both the Five Questions, K-S(535) = 0.23, p<0.001, and the CVI Questionnaire, K-S(535) = 0.17, p<0.001 the distribution of raw scores for each questionnaire was not normal. A Spearmen’s Rank correlation indicated that the raw score of the Five Questions correlated positively and significantly with the raw score of the CVI Questionnaire, *r* (533) = 0.75, 95% CI 0.70, 0.79, p<0.001. This indicates convergent validity: the raw scores of two questionnaires which are designed to measure the same construct, are related.

### Distribution of potential CVI (as identified by the questionnaires) across diagnostic groups

**Fig 2:**
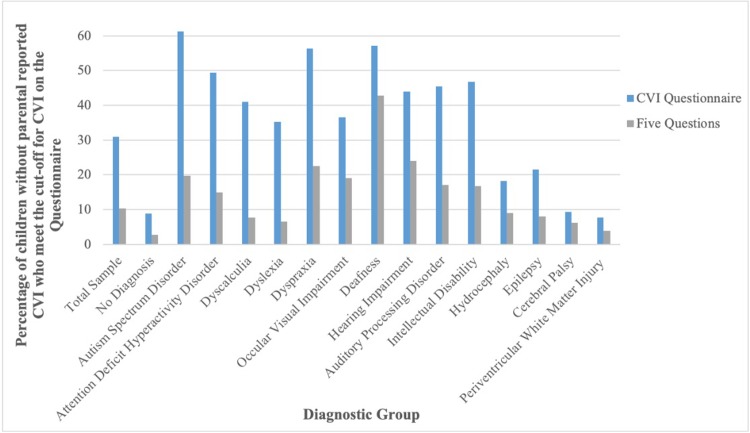
Comparison of the CVI questionnaire and the five questions at determining children with no parental-reported CVI who meet the cut-off point for CVI across diagnostic groups.

Fig 2 depicts children with potential CVI: they do not have a parental reported CVI diagnosis, but meet the cut-off for CVI on the questionnaires. Of the neurodevelopmental disorders (ASD, ADHD, ID, Dyscalculia, Dyslexia and Dyspraxia), between 6.59% and 22.53% of children were identified by the Five Questions (the more conservative questionnaire) as potentially having CVI. Many more children met the cut-off for CVI on the CVI Questionnaire than the Five Questions, however, both questionnaires were very similar with respect to the diagnostic categories which they identify as being associated with the highest percentage of potential CVI. For example, for each questionnaire, having Dyspraxia, ASD or being Deaf, are in the top four diagnostic categories with the highest incidence of potential CVI.

## Discussion

### Key results

This study evaluated the validity and sensitivity of two CVI screening measures and investigated the distribution of CVI across neurodevelopmental disorders. This is the first study to conduct Factor Analysis on the Five Questions and the CVI Questionnaire and report that they have good construct validity. In what follows, we discuss the factor analysis of the CVI Questionnaire in more detail.

As previously highlighted, the names of Factors 1–4 in the CVI Questionnaire reflect similar names of constructs outlined in the original paper [39]. However, Factor 5, named ‘Processing in Multi-tasking Activities’ by the current authors, is not a construct in the original study. It nevertheless reflects a construct which is associated with CVI [61, 62]. Factors 1–4 differ slightly from the original paper with respect to the specific items in each factor. Of note is F1-Complex Neurological Problems. In the original paper [39], this factor is named ‘complex problems’ and is loosely defined as “*complex (visuomotor) abilities”* (pp. 139). In Ortibus et al. [39], this construct only contains 2 items; “*clumsy in*: *cutting*, *building stacks*, *tying shoelaces*, *making puzzles”* and *“a moving object/person attracts more attention than a stationary one”*. In the current solution, these original items do load onto F1. In the current solution F1 also contains an additional 28 items. They relate to multiple neurological functions, not just visuomotor functions, for example, execution of movement, olfactory, touch and auditory processing, as well as visual processing. It is not uncommon for children with CVI to have complex neurological impairments beyond that of vision [63], hence, this was named ‘Complex Neurological Problems’.

An additional deviance from the constructs proposed by the original paper is F2 (Dorsal and Ventral Stream Functions). In Ortibus et al. [39], these were distinct factors. In the present study, these are combined into a single factor, reflecting research studies which demonstrate that both streams are frequently impaired [7, 14]. A further difference between Ortibus et al. [39] and the current factor structure is that one item, “*often stares at light sources”*, did not load onto any factors. This may be because staring at light sources requires several variables to be in place. For example, children may require sufficient acuity to see the light source, as well as significant attention processes in order to attend to the light. Furthermore, the question may have been difficult to interpret: it is not clear how long a stare is and what exactly is defined as a light source. Nevertheless, the factors in the current analysis reflect constructs similar to those in the original paper [39], highlighting that the CVI Questionnaire has construct validity.

As well as construct validity, this is also the first to show that the questionnaires have high internal consistency (*α* ≥ 0.9) and high convergent validity (*r* = 0.75). Additionally, it is the first study to replicate Ortibus et al. [39] and Dutton et al. [38]: both the Five Questions and CVI Questionnaire are predictive and accurately identify those with a CVI diagnosis, with sensitivity values of 81.7% and 96.2%, respectively. Thus, both questionnaires show promise as effective screening questionnaires.

Moreover, this is the first study to highlight that in comparison with typically developing children, there are a large proportion of children with neurodevelopmental disorders who have either reported CVI (23%-38.92%), or who are being identified by the questionnaires as potentially having CVI (6.59%-22.53%).

### Strengths and limitations

A limitation of this study is the reliance on parental report. It cannot be assumed that parents who attest to their child having CVI do so on the basis of a diagnostic test, therefore, it is possible that some incidences of reported CVI are erroneous. Given the large sample size, we suggest that this potential error will be small enough that it will not affect the veracity of the results, but the criterion validity of the questionnaires must still be interpreted with caution. Regression analysis indicated that meeting the questionnaire cut-offs was a significant predictor of having a reported CVI diagnosis. This analysis assumes that the children in this sample without a reported CVI diagnosis do not have undiagnosed CVI. We do not know this. The only thing one can be certain of is the sensitivity values; not specificity values or rates of false positives. This is not too problematic for the assertion that the CVI Questionnaire is predictive, as Ortibus et al. [39] analysed specificity and reported an acceptable level. However, for the Five Questions, there are no reported specificity values [38], making it possible that the Five Questions overestimates CVI in children. This caveat should be considered in the context of studies which place great emphasis on maximising sensitivity so that children are not missed [64].

The accepted standard for sensitivity values of screening questionnaires of 70–80% [65], was exceeded by both questionnaires in this study. A high sensitivity rate has been associated with lower specificity and an increased false positive rate [66]. However, with regard to developmental and behavioural disorders, it is suggested that the cost of unnecessary referrals is 99% less than that of children not being identified and missing out on intervention [65, 67]. Therefore, the high sensitivity values of both questionnaires support their utility as screening questionnaires.

A strength of this study is the large sample size (N = 535), with many diagnoses being well represented. As an initial investigation of the distribution of CVI across neurodevelopmental disorders, the group sample sizes for many of the neurodevelopmental disorders in this study are larger than many of the condition-specific studies detailed in the introduction e.g. [12, 15, 61, 68]. This sample was not randomly recruited, however, and significantly larger group sample sizes are required before a reliable indication of the prevalence of CVI across neurodevelopmental disorders can be identified. Furthermore, an associated limitation is the lack of standardisation for each questionnaire across diagnoses.

With regard to diagnostic accuracy, CVI may be difficult to differentiate from several neurodevelopmental disorders, as behavioural indices often overlap [69] and several studies have detailed how CVI could account for each of the diagnostic criteria for ADHD, ASD, Dyslexia and Dyspraxia [25, 69]. For example, one of the items of the CVI Questionnaire is, “*abandons play activity quickly*”, which is relatively common in children with Intellectual Disabilities [70] or Attention Deficit Hyperactivity Disorder [71], due to attentional difficulties. Similarly, one of the items of the Five Questions, “*finds copying words time consuming or difficult”*, is common in children with Dyslexia [72]. Initial observation of the Social Communication Questionnaire, SCQ [64, 73], a commonly used screening questionnaire for Autism Spectrum Disorder, suggests that 15% of the questions on the SCQ overlap with items of the CVI Questionnaire (S3 Table), for example, both questionnaires contain items relating to eye gaze, facial expressions, attending to voices and directing attention. It is plausible that these questionnaires may not be identifying specific aspects of CVI, but aspects of the neurodevelopmental disorders, therefore, it would be prudent to consider discriminant validity. Other items are more specific to CVI only, for example, “*does not see level differences”* and “*cannot find teddy bear (or equal) amongst other toys*” are distinctive features of optic ataxia and simultanagnosia, respectively [74]. In the current study, sensitivity values appear relatively stable and high across diagnoses (see Fig 1: > 79% for all diagnoses on both questionnaires, except the Five Questions for ADHD, which is 65%). This suggests that they are effective in identifying CVI across groups. It remains evident, however, that future research focusing on the diagnostic overlap between neurodevelopmental disorders and CVI is warranted.

### Implications for theory

Whilst the questionnaires require further replication to ascertain the accuracy of the percentages of potential CVI across groups, a large proportion of children in this sample (23%-38.92%) have parent reported comorbid CVI and a neurodevelopmental disorder (see Table 2). The CVI literature focuses predominantly on aetiology and features [75]. In contrast to the literature pertaining to Ocular Visual Impairment [76–81], there is a relative dearth of studies investigating the developmental impact of CVI [31, 82].

Drawing on knowledge of the sequential and additive nature of development, logical hypotheses about the impact of CVI on development have been proposed [69, 82]. For example, some children with CVI have difficulties with social interaction [69, 82]. It has been proposed that this may arise from dyskinetopsia [69], a type of CVI associated with dorsal stream dysfunction. Children with dyskinetopsia struggle to process fast moving facial expressions [82], potentially impacting their ability to attend to the non-verbal socio-communicative cues which are evoked through these facial expressions and eye gazes [69]. This is thought to result in atypical interactions and responses to others’ emotions [69]. However, this hypothesis has yet to be substantiated with empirical investigation. In the current study, of the 147 children with ASD, 23.81% have parent reported CVI and a further 19.73% potentially have CVI. Indeed, other studies have highlighted that many children with ASD have CVI, specifically dorsal stream dysfunction [83–88]. Like those with CVI, children with ASD also have a social interaction impairment and struggle to interpret eye gazes [89]. It is thought that this is due to impaired social motivation [90]. Given the aforementioned dyskinetopsia hypothesis, it is possible that in those with comorbid CVI and ASD, the social interaction impairment instead arises due to dyskinetopsia.

A more substantiated hypothesis suggests that CVI plays a causal role in the development of Dyslexia [91–93]. Of the 91 children in the current study with parental reported dyslexia, 26% have parent-reported CVI and a further 7% are identified by the questionnaires as potentially having CVI. Dorsal stream deficits, such as impaired visual attention shifting, eye movement control and coherent motion processing, have been reported in children with Dyslexia [94–97]. It is of note that attention shifting is correlated with phonological awareness [98], a key mechanism involved in the development of reading [98–100]. Additionally, a study of 4512 children found that the greater the difficulty a child has in activities involving the dorsal stream, the lower their attainment in reading [101]. These studies align with the hypothesis that a dorsal stream impairment may be present in children with dyslexia [93, 97, 98]. Whether or not it plays a causal role is debated [102–104], as not all children with Dyslexia show dorsal stream impairments [96; 103], others have impairments that cannot be explained by a dorsal stream deficit [102] and the exact nature of the processes involved between the dorsal stream impairment and possible development of Dyslexia is yet unclear [102].

Given the hypothesised impact of CVI on the emergence of neurodevelopmental disorders it is imperative that the developmental trajectories of children with CVI are further investigated. If CVI potentially plays a causal role in the emergence of neurodevelopmental disorders, due to features such as impaired visual attention shifting, eye movement control and motion processing, then this only further emphasises the importance of early screening and timely intervention.

### Implications for practice

The current study results indicate that the Five Questions and CVI Questionnaire are sensitive screening tools. In terms of respondent acceptability, parents in this study preferred the Likert scale format of the Five Questions to the dichotomous CVI Questionnaire. The Five Questions is shorter than the CVI Questionnaire, thus has the potential benefits of reduced reader fatigue and increased response reliability [105, 106]. Unlike the CVI Questionnaire, the Five Questions has no overlapping items with the Social Communication Questionnaire for ASD (S3 Table), thus potentially has better discriminant validity than the CVI Questionnaire, at least for children with ASD. These factors suggest that the Five Questions may be preferable for larger scale screening studies.

On the other hand, the CVI Questionnaire has much higher sensitivity than the Five Questions; arguably a more important consideration for screening studies than length or format e.g. [105]. The CVI Questionnaire is also relatively short and could be converted into a Likert scale e.g. [107, 108]. Unlike the CVI Questionnaire, the Five Questions only contains questions which refer to dorsal stream dysfunction, not ventral stream dysfunction, leading to the possibility that some children with CVI could be missed when screened with the Five Questions. Dorsal stream dysfunction is reportedly the most common type of CVI observed in children [28]. It is suggested that this is due to the inherent vulnerability of the posterior parietal lobes [28], in which the three arteries, which provide blood to either side of the brain, converge. The posterior parietal lobes are therefore particularly susceptible to damage from reduced oxygen, blood flow and glucose supply [28]. As such, it is likely that that most children with CVI would be identified by the Five Questions.

Ventral stream dysfunction can be present without an affected dorsal stream e.g. [109]. However, empirical studies comparing the prevalence of different types of CVI have not been conducted, so the prevalence rate of ventral stream dysfunction, in the absence of dorsal stream dysfunction, is unknown. Whilst the aforementioned limitations regarding the cross-diagnosis sensitivity remain, we suggest that the CVI Questionnaire would be preferable for use in large screening studies. Upon identification of CVI by either questionnaire, follow up detailed history taking and assessment of both ventral and dorsal stream functions would be required to ascertain the exact nature of the child’s CVI.

### Future directions

Many future directions have been highlighted throughout this discussion, from establishing specificity values for the Five Questions, to ascertaining the ability of the screening measures to discriminate between neurodevelopmental disorders which are behaviourally similar to CVI, as well as the need to study CVI from a developmental perspective. An additional future direction could be to consider the impact of Ocular Visual Impairment on the screening measures. For example, if a child has undetected and uncorrected visual acuity problems, without CVI, it is possible that they too, may “*have difficulty seeing something which is pointed out in the distance*” (Five Questions) or “*need more time than you’d expect to look at an object”* (CVI Questionnaire). The majority of respondents in this study were unable to provide visual acuity figures for their children, therefore this could not be controlled for in subsequent analyses. Future studies could consider the screening measures’ ability to discriminate between Ocular Visual Impairment and Cerebral Visual Impairment.

## Conclusion

The current study demonstrates that the Five Questions and CVI Questionnaire have good convergent validity, internal consistency and a reliable factor structure and may therefore be suitable as screening tools. We suggest that the CVI Questionnaire may be preferable to the Five Questions for large scale screening studies, however the Five Questions may potentially provide more accurate results in children with ASD. The study furthermore highlights that reported or potential CVI is evident in a large proportion of children with neurodevelopmental disorders. Whilst further research is required, these preliminary results align with assertions made by other researchers and emphasise the need to screen for CVI in children with Additional Support Needs, particularly those with neurodevelopmental disorders.

## Supporting information

S1 Dataset Data. (XLSX)

S1 Fig Loess lines five questions. (DOCX)

S2 Fig Scree plot five questions. (DOCX)

S3 Fig Scree plot CVI questionnaire. (DOCX)

S1 File Survey. (DOCX)

S1 Table Factor loadings five questions. (DOCX)

S2 Table Factor loadings CVI questionnaire. (DOCX)

S3 Table Overlap with social communication questionnaire. (DOCX)
