# Supplementary figures and images for: Towards population screening for Cerebral Visual Impairment: Validity of the Five Questions and the CVI Questionnaire
Source: PLoS One. 2019 Mar 26;14(3):e0214290. doi: 10.1371/journal.pone.0214290 (PMC6435113; doi:10.1371/journal.pone.0214290)

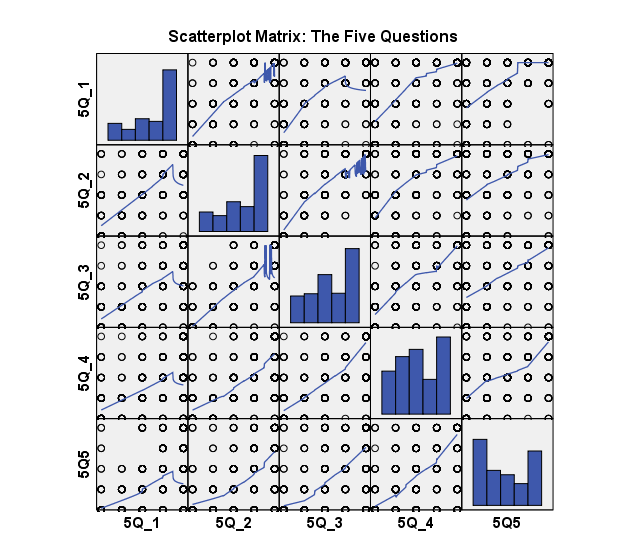

Supplement: S1 Fig — (DOCX) [file pone.0214290.s002.docx]

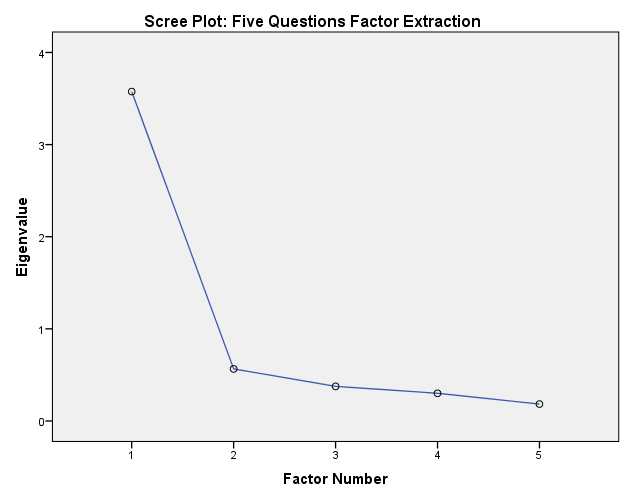


Point 1: 1 Factor

Supplement: S2 Fig — (DOCX) [file pone.0214290.s003.docx]

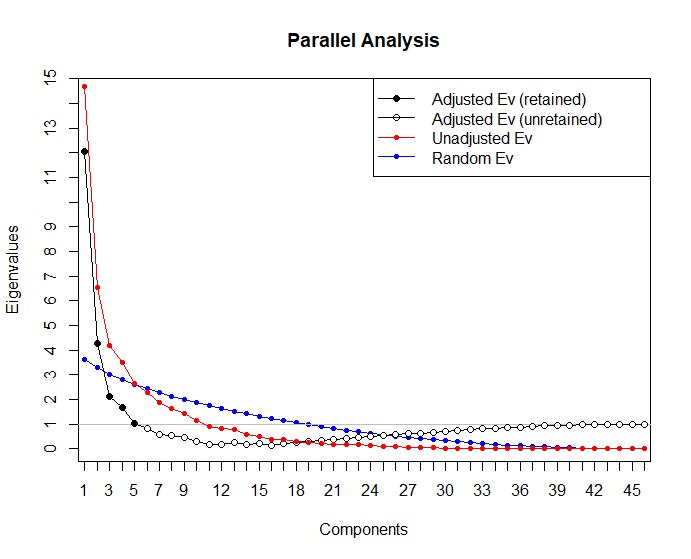


Point 1: 5 Factors

Supplement: S3 Fig — (DOCX) [file pone.0214290.s004.docx]
