# Supplementary material for: Towards population screening for Cerebral Visual Impairment: Validity of the Five Questions and the CVI Questionnaire
Source: PLoS One. 2019 Mar 26;14(3):e0214290. doi: 10.1371/journal.pone.0214290 (PMC6435113; doi:10.1371/journal.pone.0214290)
Supplement: S1 File — (DOCX) [file pone.0214290.s005.docx]

1. Child’s Date of Birth: _________ (DD/MM/YYYY)
2. Sex: □ Male □ Female

|  | Primary | Secondary |
| --- | --- | --- |
| mainstream |  |  |
| mainstream with a classroom assistant |  |  |
| special school |  |  |
| ‘special class’ in mainstream |  |  |
| home schooled |  |  |

1. Where does/did your child attend primary and secondary school? (please tick)
2. Combined yearly parental income, before tax (optional): __________
3. Please circle the statement below which best describes your child:
   - A twin
   - A triplet
   - A quadruplet
   - A multiple birth – more than 4 babies
   - None of the above – my child was a single birth
4. After how many weeks of pregnancy was your child born? _________
5. Birth weight: ___________ (lbs/ounces) OR __________ (kilograms/grams)
6. Does your child have any of the following? (tick all that apply) (For definitions see attached leaflet at end)

| - Autism Spectrum Disorder (ASD) - Attention Deficit Hyperactivity Disorder (ADHD) - Deafness - Hearing Impairment - Auditory Processing Disorder | - Dyscalculia - Dyslexia - Dyspraxia - Learning/Intellectual Disability - Down Syndrome - Fragile X Syndrome - William’s Syndrome | - Hydrocephalus - Epilepsy - Cerebral Palsy - Periventricular White Matter Injury - Total Blindness - Ocular Visual Impairment - Cerebral Visual Impairment - None of the above |
| --- | --- | --- |
| - Other (please give details): _____________________________________________   ___________________________________________________________________ | | |

1. Has your child ever had an eye exam? □ Yes □ No
2. Has your child been prescribed glasses? □ Yes □ No
   - If yes: - Do they wear them: □ all the time □ occasionally □ not at all

- What is their visual acuity (if known): ______________

| 11) Dutton et al., 2010 PLEASE TICK | Always | Often | Sometimes | Rarely | Never |
| --- | --- | --- | --- | --- | --- |
| Does your child have difficulty:  Walking down stairs? |  |  |  |  |  |
| Seeing things which are moving quickly, such as small animals? |  |  |  |  |  |
| Seeing something which is pointed out in the distance? |  |  |  |  |  |
| Locating an item of clothing in a pile of clothes? |  |  |  |  |  |
| Does your child find copying words or drawings time-consuming and difficult? |  |  |  |  |  |

| 13) Ortibus et al., 2011 PLEASE TICK ALL THE BOXES THAT APPLY TO YOUR CHILD | √ |
| --- | --- |
| Absent eye contact |  |
| Cannot focus on persons nor objects |  |
| Tilts head to look at objects |  |
| Often stares at light sources (lights, open windows) |  |
|  |  |
| Falls frequently over clearly visible objects |  |
| Does not find his/her toy when he/she drops it |  |
| Bumps easily into something |  |
| Pays attention only to objects in the centre of his/her visual field |  |
|  |  |
| Cannot keep looking at objects or persons |  |
| Attention is fluctuating from moment to moment and from day to day |  |
| Abandons his/her play activity quickly |  |
| Needs more time than you’d expect to look at an object |  |
| Does not look spontaneously at an object/ does not explore the room spontaneously |  |
| Needs encouragement to look at an object, explore the room |  |
| More toys perturb visual attention |  |
| Objects are looked at from a short distance |  |
| Sits right in front of the television |  |
|  |  |
| Scared or restless in unfamiliar environment (shop, street,..) |  |
| Does not find his/her parents when they stand further away |  |
| Clings to parents in an unfamiliar environment |  |
|  |  |
| Does not recognize everyday objects such as an apple, bike, house, ball,… |  |
| Recognizes familiar objects only when they are drawn in colour |  |
| Recognizes persons rather by listening to their voice, watching their posture than by looking at their faces |  |
| Does not understand facial expressions (mad, sad, glad,..) |  |
| Does not find his/her way to the classroom/ in his/her house (familiar environments) |  |
|  |  |
| Does not see level differences (stairs,..) |  |
| Cannot take the chocolate spread from the breakfast table without difficulty |  |
| Looks away when he/she takes the chocolate spread from the table |  |
| Has no interest for simple pictures |  |

| 13) CONT.. PLEASE TICK ALL THE BOXES THAT APPLY TO YOUR CHILD | √ |
| --- | --- |
| Has no interest for complex pictures |  |
| Looks only at details of a picture |  |
| Cannot find his/her teddy bear (or equal) amongst other cuddly animals |  |
| Does not find the chocolate spread on the table |  |
| Does not find/recognize familiar persons in a crowd |  |
| Cannot estimate distances |  |
|  |  |
| Clumsy in: cutting, building stacks, tying shoelaces, making puzzles |  |
| A moving object/person attracts more attention than a stationary one |  |
|  |  |
| Reacts faster to sound than to visual stimuli |  |
| Manipulates an object rather than to look at it |  |
| Always puts objects, toys in his/her mouth |  |
|  |  |
| Cannot play memory games |  |
| Stops activity when there is too much to look at (eg in a busy environment) |  |
| Is generally anxious |  |
| Does not do his/her best for tasks for which he/she needs to look carefully |  |
| I often wonder: does he/she not want to look at things or is he/she not able to? |  |
| He/she tries to compensate by talking a lot |  |

Anything you’d like to add: _________________________________________________________________________________________________________________________________________________________________________________________________________________________________

Definition for above questions.

- *Autism Spectrum Disorder:* difficulty with social interaction and social communication as well as having restricted, repetitive patterns of behaviour, interests or activities.
- *Attention Deficit Hyperactivity Disorder:* mild to severe level of difficulty due to inattention and/or hyperactivity and impulsivity.
- *Deafness:* damage to the ear/hearing nerve – no hearing at all.
- *Hearing Impairment:* damage to the ear/hearing nerve – some hearing.
- *Auditory Processing Disorder:* hearing impairment due to damage to brain – the brain has difficulty processing and interpreting sound, even though it receives it (or ‘hears’ it) through the ears.
- *Learning/intellectual disability:* mild to profound level of difficulty with everyday activities, learning new skills and understanding complicated information.
- *Dyscalculia:* specific learning difficulty in basic maths.
- *Dyslexia:* specific learning difficulty in reading and writing.
- *Dyspraxia:* something which affects physical coordination and causes a child to appear to move clumsily.
- *Hydrocephalus:* a build-up of fluid on the brain.
- *Periventricular white matter injury:* damage to white matter in the brain. Those with it often have difficulties with motor control.
- *Total Blindness –* damage to the eyes – no vision at all
- *Ocular Visual Impairment –* damage to the eyes – some vision
- *Cerebral Visual Impairment –* visual impairment due to damage to the brain – the brain has difficulty processing visual information, even though it receives it (or ‘sees’ it) through the eyes.
