# Supplementary material for: Towards population screening for Cerebral Visual Impairment: Validity of the Five Questions and the CVI Questionnaire
Source: PLoS One. 2019 Mar 26;14(3):e0214290. doi: 10.1371/journal.pone.0214290 (PMC6435113; doi:10.1371/journal.pone.0214290)
Supplement: S1 Table — (DOCX) [file pone.0214290.s006.docx]

|  | Factor 1: Dorsal Stream |
| --- | --- |
| 5Q_1: See things moving quickly | 0.88 |
| 5Q_2: See things pointed in distance | 0.86 |
| 5Q_3: Walk downstairs | 0.81 |
| 5Q_4: Find item in pile of clothing | 0.78 |
| 5Q_5: Copy words or drawing = time consuming | 0.68 |

S4 Table 5Qs Factor Loadings
