# Supplementary material for: Towards population screening for Cerebral Visual Impairment: Validity of the Five Questions and the CVI Questionnaire
Source: PLoS One. 2019 Mar 26;14(3):e0214290. doi: 10.1371/journal.pone.0214290 (PMC6435113; doi:10.1371/journal.pone.0214290)
Supplement: S2 Table — (DOCX) [file pone.0214290.s007.docx]

S6 Table Factor Loadings CVI Questionnaire

|  | F1: Complex Neurological Problems | F2: Dorsal and Ventral Stream Functions | F3: Visual Attention | F4: Influence of a Familiar Environment on Vision | F5: Parallel Processing in Multi-tasking activities. |
| --- | --- | --- | --- | --- | --- |
| **Cronbach’s Alpha** | **0.94** | **0.89** | **0.89** | **0.75** | **0.67** |
| Looks away when he takes the chocolate spread from the table | 0.86 |  |  |  |  |
| Clumsy in: cutting, building stacks, tying shoelaces, making puzzles | 0.86 |  |  |  |  |
| Bumps easily into something | 0.85 |  |  |  |  |
| Does not do best for tasks which he needs to look carefully | 0.78 |  |  |  |  |
| He tries to compensate by talking a lot | 0.76 |  |  |  |  |
| Falls frequently over clearly visible objects | 0.72 |  |  |  |  |
| Cannot take the chocolate spread from the breakfast table without difficulty | 0.71 | 0.4 |  |  |  |
| Always puts objects/toys in mouth | 0.71 |  |  |  |  |
| Manipulates objects rather than to look at it | 0.63 |  |  |  |  |
| Looks only at details of pictures | 0.62 |  |  |  |  |
| Cannot find teddy bear (or equal) amongst other cuddly toys | 0.61 |  |  |  |  |
| More toys perturb visual attention | 0.57 |  |  |  |  |
| Does not find chocolate spread on table | 0.57 | 0.44 |  |  |  |
| I often wonder: does he not want to look or is he not able to? | 0.55 |  | 0.34 |  |  |
| Objects are looked at from a short distance | 0.54 |  | 0.33 |  |  |
| Absent Eye Contact | 0.53 |  | 0.38 |  |  |
| Pays attention only to objects in the centre of visual field | 0.51 |  |  |  |  |
| Cannot estimate distances | 0.51 | 0.48 |  |  |  |
| Attention is fluctuating from moment to moment and from day to day | 0.49 |  |  |  | 0.61 |
| Sits right infront of the TV | 0.47 |  |  |  |  |
| Does not find/recognise familiar persons in a crowd | 0.44 | 0.67 |  |  |  |
| Does not see level differences | 0.42 | 0.57 |  |  |  |
| Does not find his toy when he drops it | 0.42 | 0.37 |  |  |  |
| Has no interest for complex pictures | 0.40 | 0.44 |  |  |  |
| Is generally anxious | 0.38 | -0.31 |  | 0.54 |  |
| Stops activity when there is too much to look at (e.g. in a busy environment) | 0.36 |  |  | 0.36 |  |
| Reacts faster to sound than visual stimuli | 0.34 | 0.54 |  |  |  |
| A moving object/person attracts more attention than a stationary one | 0.32 |  |  |  |  |
| Needs more time than you’d expect to look at an object | 0.31 |  | 0.35 |  |  |
| Cannot keep looking at objects or persons | 0.30 |  | 0.35 |  |  |
| Does not recognise everyday objects such as an apple, bike, house, ball… | -0.51 | 1.08 |  |  | 0.36 |
| Does not find his/her parents when they stand further away |  | 0.68 |  |  |  |
| Recognises familiar objects only when they are drawn in colour |  | 0.62 |  | 0.41 |  |
| Recognises persons rather by listening to their voice, watching their posture than by looking at their faces |  | 0.74 |  |  |  |
| Does not find his way to classroom, in his house (familiar environments) |  | 0.56 |  |  |  |
| Does not look spontaneously at an object, does not explore room spontaneously |  |  | 0.93 |  |  |
| Needs encouragement to look at an object/explore room |  |  | 0.85 |  |  |
| Scared or restless in unfamiliar environment |  |  |  | 0.66 |  |
| Clings to parent in unfamiliar environment |  |  |  | 0.82 |  |
| Abandons his play activity quickly |  |  |  |  | 0.64 |
| Cannot focus on persons nor objects |  | 0.35 | 0.38 |  |  |
| Tilts head to look at objects |  | 0.40 |  |  |  |
| Does not understand facial expressions (mad/sad/glad…) |  |  | 0.33 |  |  |
| Has no interest for simple pictures |  | 0.34 | 0.48 |  |  |
| Cannot play memory games |  | 0.49 |  |  | 0.44 |
|  | | | | | |

^1^*Note*: As oblique rotation was carried out, factor loadings reflect regression coefficients and can be a value >1
