# Supplementary material for: Towards population screening for Cerebral Visual Impairment: Validity of the Five Questions and the CVI Questionnaire
Source: PLoS One. 2019 Mar 26;14(3):e0214290. doi: 10.1371/journal.pone.0214290 (PMC6435113; doi:10.1371/journal.pone.0214290)
Supplement: S3 Table — (DOCX) [file pone.0214290.s008.docx]

S8 Table Social Communication Questionnaire (SCQ) and Items which overlap with the CVI Screening Questionnaires

| SCQ | CVI Questionnaire | Five Questions |
| --- | --- | --- |
| Inappropriate Facial Expressions  Range of Facial Expressions | Does not understand facial expressions |  |
| Attention to voice | Recognises persons rather by listening to their voice, watching their posture than by looking at faces. |  |
| Eye gaze | Absent eye contact |  |
| Response to other children’s approaches |  |  |
| Social smiling |  |  |
| Showing and directing attention | Needs encouragement to look at an object, explore the room.  Attention is fluctuating from moment to moment and from day to day. |  |
| Use of other’s body | Clings to parents in an unfamiliar environment. |  |

*Note:* The SCQ has 40 items.
